# Practical aliquoting of flowering plant genomes

**DOI:** 10.1186/1471-2105-14-S15-S8

**Published:** 2013-10-15

**Authors:** Chunfang Zheng, David Sankoff

**Affiliations:** 1 Department of Mathematics and Statistics, University of Ottawa, 585 King Edward Avenue, Ottawa, Canada, K1N 6N5

## Abstract

We pose the problem of dissecting an ancient polyploid genome into its constituent subgenomes despite fragmentation and noise caused by genome rearrangements and fractionation of multi-copy genes. We formulate this in terms of decomposition into "defective" *k*-partite graphs, distinguished by location within the genome. We devise and implement a clustering heuristic for solving realistic instances of the problem. An unusual focus of our method is the focus on prioritizing gene density or lack of gaps in the assembly of fragments into larger regions, rather than maximizing the number of genes. We validate the method against the grape genome in which the ancient core eudicot triplication is readily detectible and is already well known. We then analyze the tomato genome, whose proposed status as a descendant of a more recent *Solanum *hexaploid is controversial, and confirm this proposal. The solution reveals unexpected information about the evolution of the tomato.

## Introduction

Around 200 Mya, a whole genome duplication (WGD) was fixed in some gymnosperm, or other seed plant [1] as yet unidentified [2], and within perhaps ten or twenty million years, a short time in geological terms, its descendants had evolved the complex reproductive structure known as the flower. Thanks to this innovation, flowering plants (a phylum or division variously known as the angiosperms or the Magnoliophyta), diversified and expanded into almost all ecological niches on land and in many partially or largely aquatic locales, eventually, by the end of the Cretaceous period, dominating all plant life in these contexts.

WGD is distinguished from the classical term "tetraploidization" for the combination of two genomes, in that WGD involves the re-diploidation of the meiotic process, and fixation of the new form as a characteristic of a separate species. WGD events recur in lineages on the scale of tens of million years intervals, but the adaptive genetic resources that doubling confers is often followed by rapid radiation of diverse descendants, so that published genome sequences of flowering plants show that additional WGD events (loosely including also triplication, i.e., fixed, re-diploidized, hexaploidization, and higher order combinations) occurred in almost all lineages leading to modern species, often twice or three times, sometimes up to four or five events. One of the most significant of these events followed the hexaploidization of the ancestor of the core eudicots, which include the majority of all flowering plant species, an event first discovered in 2007 with the sequencing of the grape genome [3], and confirmed for the entire rosid grouping with the cacao genome [4].

Recurrent WGD complicates the comparative genomic study of flowering plant evolution. In contrast to other evolutionary domains of comparable time depths, the problem in comparing these plant genomes or reconstructing ancestral genomes, especially at the level of gene order is not such much the order-scrambling effects of chromosomal rearrangements, nor is it the confusing effects of high levels of paralogy. Instead it is the effects of "fractionation" [5], variously termed in the literature as "reciprocal gene loss" [6], "interleaving" [7,8], or with a different emphasis, "double synteny" [9,10].

After WGD, the traces of the original two (or more) genomes, called "(homeologous) subgenomes", may be evident in the genome though large numbers of duplicate genes that are not tandem pairs, but arrayed as largely similar "homeologous" fragments in two or more different chromosomes.

The term "aliquoting" was originally coined [11,12] for the purely theoretical problem of dissecting of the diploid genome of a descendant of an ancient hexaploid, octoploid, etc. into its constituent subgenomes, where no genes have been lost in the course of evolution by chromosomal rearrangement. This was a generalization of the "halving problem" for ancient tetraploids [13]. In this paper we use the notion of aliquoting to apply more broadly to the inference of subgenomes, or portions of them, in the realistic situation of descendants of WGD events where fractionation has eliminated most of the duplicate genes so that the genomes consist mostly of single copy genes.

We first formalize the aliquoting problem is the context of rearrangement and fractionation. We then motivate and present a heuristic greedy algorithm to search for a solution. This method is based on principles that were first applied systematically to the genome of the core eudicot, grape, in 2007 [3]. These principles are essential to a correct analysis but are not explicitly incorporated or prioritized in existing more general methods for the treatment of paralogy. Next, this algorithm is shown to correctly aliquot the grape genome into seven sets of triplicated chromosomes or large chromosomal fragments, which had originally been discovered by manual means. Then we apply the method to the difficult case of a more recent WGD affecting both the tomato and potato genomes [14], which have been considered as descending from a triplication or partial triplication in the *Solanum *lineage. Our results reveal some previously unknown differences among the three subgenomes contributing to the tomato genome, and inform a discussion of the dynamics of the WGD event.

## Definitions

**Figure 1:**
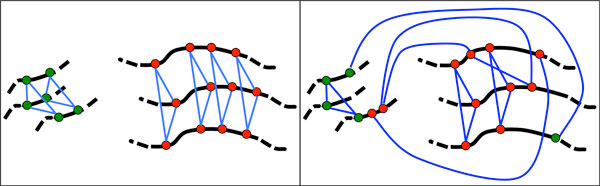
**Left: part of a 3-partite graph formed of 3-cliques, representing a newly formed hexaploid**. Red dots represent vertices in three homeologous chromosomes, green dots another three chromosomes, and blue lines are the graph edges connecting paralogous genes. The black lines represent triples of chromosomes with parallel linear ordering. (These lines are not edges in the 3-partite graph.) Right: same genome after some genome rearrangement and gene loss.

Aliquoting has two graph theoretical aspects, reflecting two independent characteristics of genome organization. The first, *k-partition*, has to do with homology among genes within a 2*k*-ploid genome, more particularly the *k*-way paralogies set up after a whole genome undergoes 2*k*-ploidization. The second, *k-synteny*, involves gene positions on the chromosome. After the 2*k*-ploidization of a genome originally containing *n *genes on *C *chromosomes, each of the *kn *genes in the new *kC*-chromosome genome can be considered a vertex in a *k*-partite graph connected by *k − *1 edges to exactly one gene (its paralog) in each other part of the graph, thus forming *n *disjoint *k*-cliques. This is *k*-partition. In addition each vertex is linearly ordered with respect to some subset *χ *of the other vertices - *with no edges (paralogies) among them *- representing one of the *kC *chromosomes, and these subsets are disjoint. The orderings are reflected exactly within each of *k − *1 other chromosomes, called *homeologous chromosomes*, containing one paralog of each of the genes in *χ*. The parallel orderings constitute *k*-synteny. This is illustrated in Figure 1 for *k *= 3.

The paralogy graph and the homeology subsets representing an initially 2*k*-ploid genome evolve over time through chromosomal rearrangement and duplicate gene fractionation, introducing "defects" into both the *k*-partition and the *k*-synteny. The rearrangements disrupt the linear order of the chromosomes, and may also involve the exchange of vertices between two subsets (chromosomes). Moreover, most of the vertices may simply be deleted from the graph, representing gene loss and paralogy loss, although at least one gene, "single-copy", in each set of *k *paralogs must remain.

The aliquoting problem becomes: Given *k *> 2 and any graph endowed with a partition of its vertices into *n *> 0 connected components, which are cliques containing between 1 and *k *paralogous genes, and given another partition of these vertices into a number of sets each of which is linearly ordered, to try to detect the "remains" of a 2*k*-ploid, by verifying whether it is *k*-partite, or almost so, and whether some regions of largely parallel linear ordering can be detected in *k *copies respecting the paralogy. To make this statement more precise requires specifying how deviations from strict *k*-partition are penalized relative to gaps between fragments in a region compared to the given linear ordering, as well as other considerations discussed in the next section.

## The search for subgenomes

For the genome to be aliquoted, the input to our procedure is its gene order along its chromosomes, together with a partition of all the genes into sets of at most *k *paralogs, where *k *> 2 is the suspected degree of polyploidy, fixed in advance. Genes occurring only in single copies are ignored because they contain no information relevant to the choices made during aliquoting. Each paralogy set consists of at least two genes, the remaining copies having been deleted in the course of evolution. Each gene is identified by a distinct label and its only two relevant properties are its position on a specific chromosome, and the set of paralogs it belongs to.

We use the SynMap procedure in CoGe [15,16] to extract these data via a self-comparison of the genome. We assume this information is completely accurate, or very nearly so, both with respect to gene order and paralogy assignment. The largest source of error may be the contamination of the paralogy data due to a WGD with data from an older or younger WGD. This can be controlled to a large extent in pre-processing by filtering the paralogs so that their sequence level similarity is within a suitable constrained range. This is a more precise operation when controlling for more recent WGD as its distribution of similarities will be relatively compact, while the range in similarities from older WGD is more likely to overlap with that of the WGD being aliquoted.

After filtering, for the paralogy sets that remain with more than *k *elements, in a second pre-processing step, we provisionally delete the edges representing the weakest level of sequence similarity (perhaps from older WGD), until there remain only *k *connected elements in the set.

Note that at this stage, no information about the bulk of single-copy genes is retained for the analysis.

While the paralogy relation among surviving (non-fractionated) genes can be assumed to have been constant since the polyploidization event, the gene positions have been subject to rearrangement and we can only hope to identify relatively long multiply copied regions in the *k *subgenomes.

Our procedure is essentially an agglomerative clustering algorithm producing clusters that each have at most *k *internal orderings, called *regions *representing parts of the original subgenomes. At the outset each paralogy set is considered a cluster containing one item, namely the set itself.

We use three parameters to control the agglomeration step in the algorithm, a "short gap" reward *r *> 0, a chromosome "jump" penalty *j <*0 and an "aliquoting defect" penalty *h*. A fourth parameter, threshold *t *> 0, is applied in post-processing to modify very short regions.

Some terminological distinctions: A *fragment *is a contiguous set of genes on a chromosome of the input genome. (This ignores any single-copy genes, which have already been removed from consideration.) A *region *is an ordered set of fragments, with successive fragments being separated by a *gap *of one or more genes on a chromosome, or by a *chromosome jump*, i.e., the two fragments are on different chromosomes. A *k*-tuple of regions contains *k *regions where ideally all the paralogs of all the genes are between the regions and none are within a single region. Pairs of paralogs that are exceptions to this rule are called *aliquoting defects*.

The key step in the algorithm sketched below is the iterative clustering together of two existing clusters, which are *k*-tuples of regions, to make a larger regions. The best pair of *k*-tuples to merge is determined by a score calculated by comparing the two original clusters with the potential new one. When two regions are merged, some gaps may be filled in, completely or in part, and some gaps may be created, such as between the end of one region and the beginning of the other. If the merger were to reduce the total number of gapped genes, it is assigned score *r*. If it does not reduce the total number of gapped genes, the score component due to gaps is max(0, *r − x*) where *x *is the change in total number of gapped genes in the new region. In addition there is a penalty *j *if the number of chromosomes of the input genome in the two regions being merged is less than the number in the output. Finally, if the number of aliquoting defects in the merged regions is *d *greater than that in both of the original regions, a penalty of *hd *is assessed. The score *S*(*i*_1_*, i*_2_) associated with the candidate merger of regions *i*_1 _and *i*_2 _is thus the gap component plus the chromosome component, summed across *k *paralogous regions, plus an aliquoting defect component:

(1) $$S\left(i_{1},i_{2}\right)=\underset{k\;\text{regions}}{\sum }\left[\max\left(0,r-x\right)-j\chi \left(\text{jump}\right)\right]-\text{hd}\chi \left(d>0\right),$$

where *x *= 0 if the number of gapped genes does not increase, and *χ*(jump) and *χ*(*d *> 0) are indicator functions of increased jumps and increased aliquoting defects, respectively.

Algorithm aliquote

• **Parameters: **hypothesized ploidy parameter *k *> 2, short gap reward *r *> 0, jump *j *> 0, aliquoting defect penalty *h *> 0, threshold *t *≥ 0.

• **Input: ***n *> 0 paralogy sets, each containing at most *k *genes. Genes distributed and ordered on *C*' chromosomes.

• **Output: **A number *C" ≥ *1 of *k*-tuples of regions

• **Initialization:**

- Each set of paralogs defines a *k*-tuple of regions, each region consisting of at most one fragment made up of one gene.

- For all pairs of *k*-tuples of regions, calculate their clustering score *S*.

• **while **there remain pairs of *k*-tuples of regions with *S *> 0,

- merge the pair of *k*-tuples of regions with max *S*,

- delete merged pairs and add the resulting larger *k*-tuple of regions,

- calculate the clustering score *S *of the new *k*-tuple of regions with all other *k*-tuples

• **Post-processing **If the gaps between two consecutive fragments in any region is smaller than threshold *t*, move the missing genes from their current location to fill in the gap as long as any resulting aliquoting defects in the *k*-partition are not excessive. It is preferable to set *t *to as low a value as possible if this does not cause a proliferation of very small regions.

The initialization of the coefficients requires quadratic time, but may they be stored to allow rapid search; the update step proceeds in linear time since only the coefficients involving the two clusters being combined are affected. The iteration stops when no further amalgamation has positive score, after a number of steps less than *n*, so that the total running time requirement is quadratic.

The post-processing step involves some subjective judgment about how many aliquoting defects and how many small regions are tolerable. This can of course be formalized, but it will always be dependent on the specific problem instance and to what purposes the solution will be applied.

## Grape

**Figure 2:**
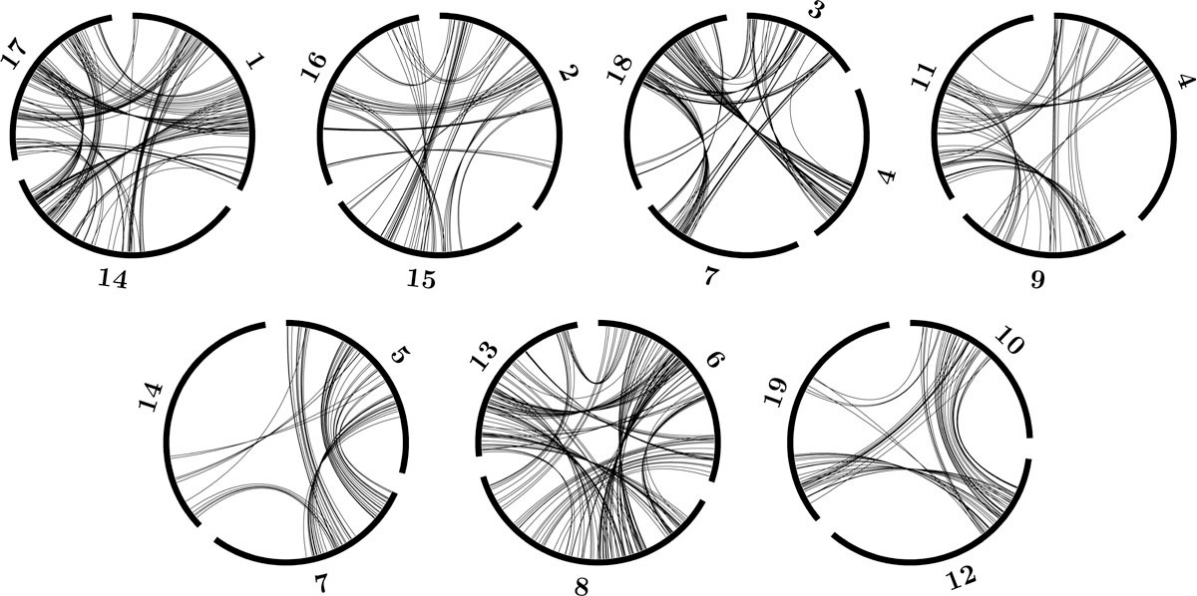
**Aliquoting of grape genome into three subgenomes by the **aliquote **algorithm**. Arcs making up the circumference of each circle represent grape chromosomes. Lines within circles represent paralogies established at the time of hexaploidization. Note that between circles defined by the algorithm, hypothesized to reflect the seven pre-hexaploid core eudicot chromosomes, there are virtually no paralogies, and within circles, no paralogies within subgenomes, and many paralogies between subgenomes. Note also that chromosomes 4, 7 and 14 appear in two circles each, reflecting chromosomal fusions in the grape lineage, while one circle is labeled by four chromosomes: 3, 4, 7, 18 because of the incorporation of fissioned parts of an ancient chromosome into chromosome 4 and 7.

The core eudicots are all descendants of an ancestral hexaploidization approximatively 125 Mya, leading within a few million years to a remarkably diverse radiation into many orders, most of which are grouped into the rosid and asterid subclasses. Among the sequenced core eudicot genomes that have been published, the grapevine [3], a rosid, is perhaps the most conservative, from the viewpoints both of sequence mutation rates and gross chromosomal structure. From the latter, the original hexaploid structure can be inferred to have involved the tripling of seven chromosomes, the grapevine conserving most of this with a handful of chromosomal fusions and fissions reducing the 21 ancestral chromosomes to 19. Figure 2 shows the results of applying our algorithm to grapevine genome paralogy data produced by comparing the genome to itself, using SynMap [16].

## Tomato

**Figure 3:**
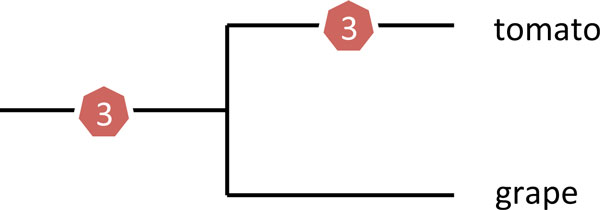
**Phylogenetic relationship of grape and tomato, showing timing of two WGD (triplications)**.

The tomato and potato genomes show clear evidence of sharing a common hexaploidization event in their history [14] long before their divergence some three million years ago. As core eudicots, these *Solanum *species also share the same WGD that we have analyzed above in the grape genome. This is illustrated in Figure 3.

**Figure 4:**
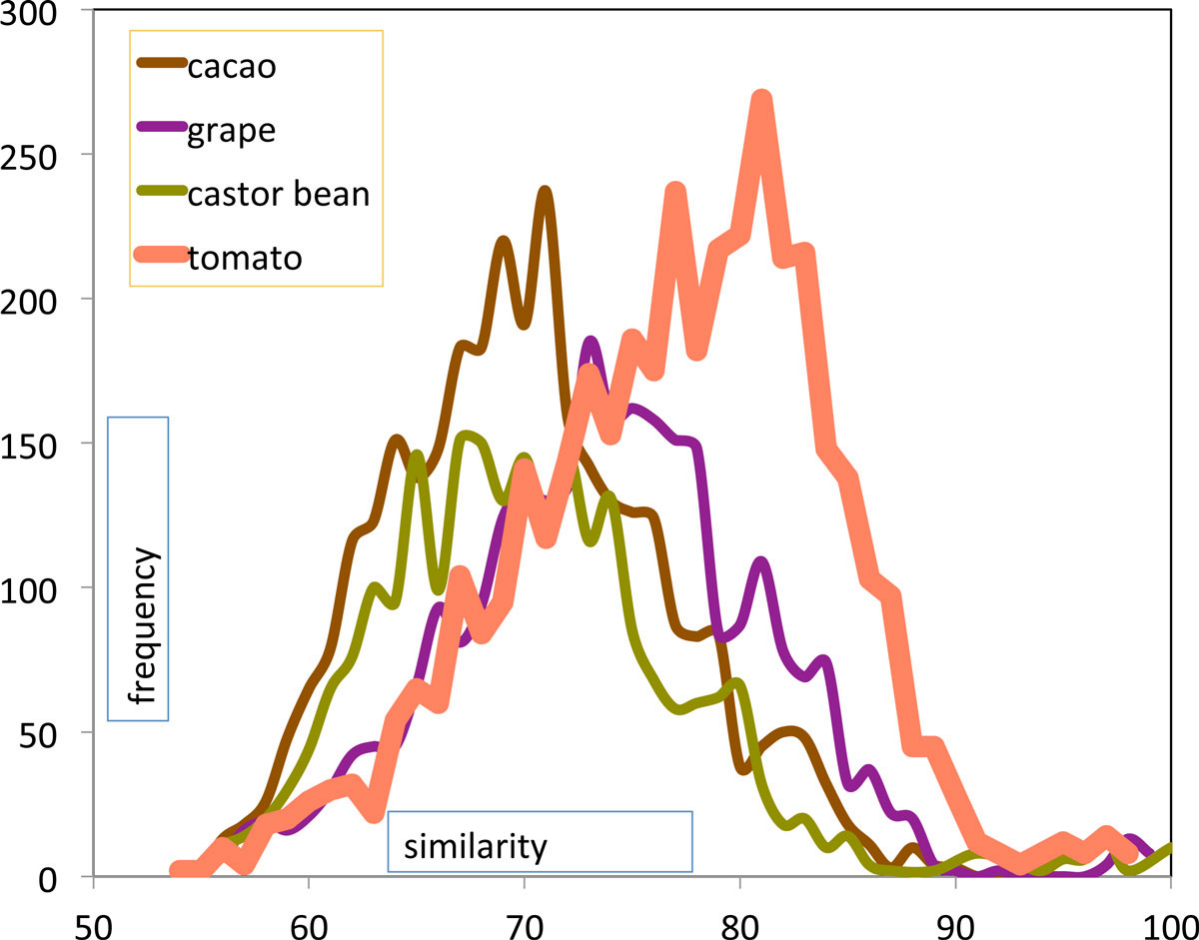
**Histograms of CoGe similarity scores of paralog pairs from core eudicot WGD and *Solanum *WGD in tomato, and in three other core eudicots unaffected by further WGD**.

Before aliquoting the tomato genome with respect to the more recent event then, we filter out as much contaminating data, namely paralogies which date from the earlier event (Figure 4). This is done with a cutoff of pairs with similarity below 72.

As is clear from the figure, the filtering step will remove the bulk of the paralogs from the earlier triplication, but leave most of the more recently created ones. This ensures that our data captures most of the information on the *Solanum *triplication, while minimizing the contamination from the core eudicot event.

### Unexpected properties of the subgenomes

**Figure 5:**
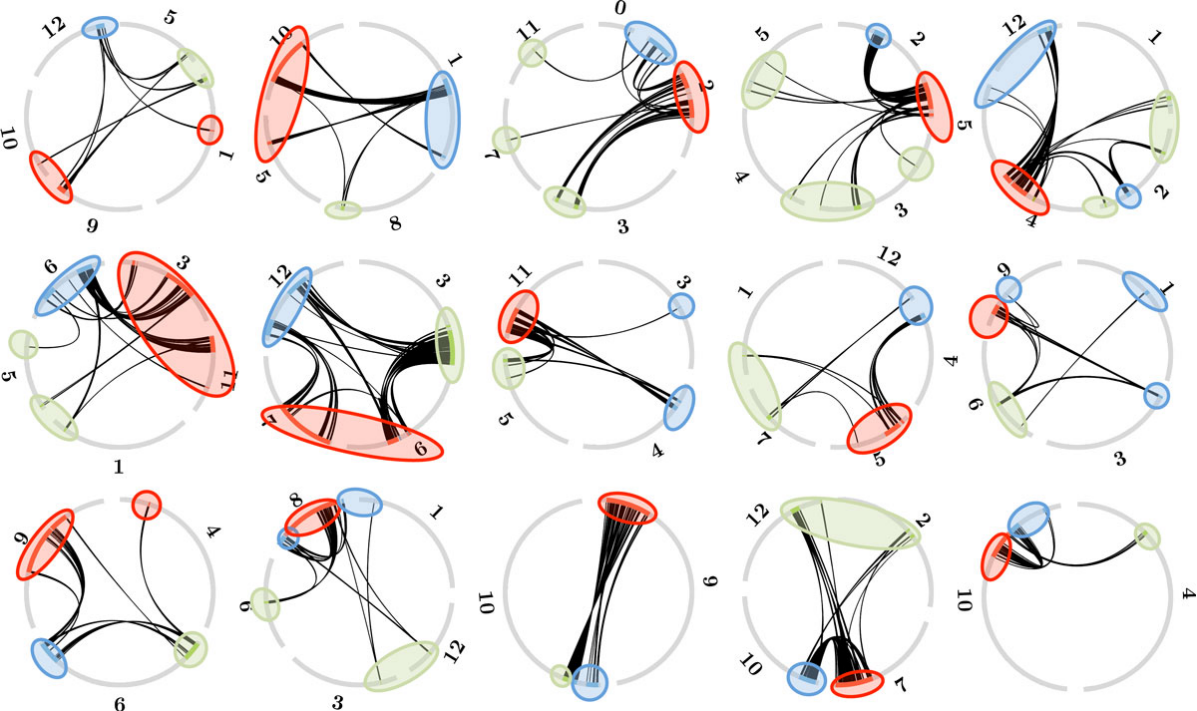
**Aliquoting of tomato genome into three subgenomes**. Note that between circles, which reflect the 15 hypothesized pre-hexaploid *Solanum *chromosomes, there are virtually no paralogies, and within circles, no paralogies within subgenomes, and many paralogies between subgenomes. Note also that several chromosomes each appear in more than one circle, reflecting chromosomal fusions in the *Solanum *lineage, and that most circles are labeled by more than three chromosomes because of the assorted amalgamation of fissioned parts of ancient chromosome.

In Figure 5, we have coloured each set of tripled regions red, blue and green according to which contained the largest, second largest, and smallest number of genes. The differential between the red regions and the others is far too great to be attributed to multinomial sampling with equal probabilities, and is reminiscent of the situation of other flowering plant WGD descendants [17], where *subgenome dominance *survives despite rearrangements breaking up and reassembling the chromosomes irrespective of their WGD origins. Thus the red regions in Figure 5 would all or mostly originate in the same subgenome at the time of hexaploidization. The dominant subgenome, early in this process, by means of regulatory and epigenetic mechanisms, depresses the expression level of the genes in the other subgenomes and facilitates their loss during fractionation (cf. the discussion in [17]).

This is a likely explanation, but does not account for another aspect of the pattern in Figure 5. We might expect, all things being equal, that the largest region be distributed among more tomato chromosomes while the smallest regions be found on only one chromosome. In fact we observe the opposite, with the smallest regions, coloured green, being spread over 1.8 chromosomes, on the average, with the regions coloured red being confined to 1.4 chromosomes, and the blue-coloured regions in between (1.53).

This observation is consistent with the hypothesis that all the red regions originate with a single subgenome that joined an original tetraploid (reflected in the blue and green regions), already considerably fractionated and rearranged. However, the blue-green tetraploidization would not have been an autoploidy event since the average sequence similarity blue-green paralogs are not significantly greater than that of red-blue or red-green paralogs.

In trying to detect triplicated regions in the tomato genome directly through automated syntenic block extraction in a self-comparison of the tomato genome, well over half of the blocks found indeed occur in triples. However, a good number only occur in pairs, leading to the suggestion that the *Solanum *hexaploidization, or its fixation, was only partial. It is quite possible for our algorithm to detect regions that are only duplicated, but nevertheless our output strongly suggests that every region is triplicated.

### The parameters of the algorithm and the properties of the solutions

Among the characteristics of an aliquoting solution that may be of interest are the proportion of genes in the input genome that are included, the number of fragments, *C*", the number of *k*-tuples of regions, and the number of "inconsistent" genes, those that cause defects in the *k*-partition as a result of the post-processing to remove small regions. By changing the parameter values, we can alter the solution, mostly by merging regions that share the same chromosomes, but whose fragments are all further apart than *r*.

**Figure 6:**
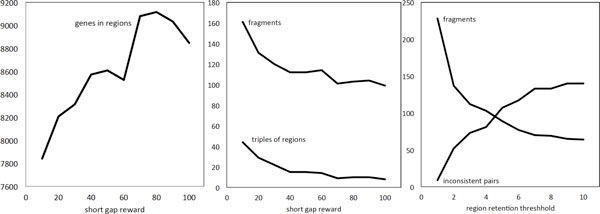
**Effects of the parameters**.

The solution in Figure 5 was calculated with *r *= 40, *j *= 15, *t *= 3. Some effects of changing *r *and *t *on the characteristics of the solution are sketched in Figure 6.

## Conclusions

From the algorithmic viewpoint, the success of our method in recruiting such a large proportion of genes to the aliquoting solution is a somewhat unexpected bounty from our preoccupation with fractionation. Given that there is no explicit attempt to favour the construction of long fragments during the clustering procedure, but only the avoidance of long gaps and the reduction of existing gaps, there is no proliferation of regions with dense fragments scattered around several tomato chromosomes; most regions are confined to one or two chromosomes. In the case of grape, that the aliquoting produced the known triples of chromosomes could be attributed to the unmistakeable patterns discovered in 2007, but the clear results in the case of tomato are obtained despite a relatively high degree of gross chromosomal rearrangement after (or during) the WGD event.

The aliquoting procedure does not take into account the single-copy genes that comprise almost 3/4 of the tomato genome. However, if we define the "span" of each aliquoted region as the chromosomal fragment(s) between the most distant genes in that region as output by the algorithm, and if we establish where each single-copy gene is located on a chromosome, within a region or outside all regions thus defined, a gratifying total of 22,000 tomato genes out of 34,000 are spanned by these non-overlapping regions.

In this work, we have not discussed the problem of finding *k *or *C*", although we did mention the controversy over whether the tomato genome is only a partial triplication. For the time being, it would seem that reference to the biological literature is still the most helpful approach. The number of chromosomes is often relatively stable within a genus or family, and the ancestral ploidy may be inferred by reference to related genomes.

This work focusing on fractionated descendants of WGD events illustrates the insights that we can obtain through the combined analysis of genome rearrangements and fractionation. The prevalence of WGD in flowering plant lineages provides strong motivation for further work in this direction.

There are other approaches to studying the evolutionary history of fractionated genomes. For example, we have been developing a "consolidation" approach which tries to "undo" the gene loss [18,19], and which produces a smaller, transformed genome, a pure polyploid with no gene loss, which can then be analyzed with classical halving or aliquoting methods. But these methods are not yet ready for application to single genomes, and still require a diploid outgroup, which is not required here.

## Competing interests

The authors declare that they have no competing interests.

## Authors' contributions

CZ and DS designed the study, carried out all the research and wrote the article.

## Declarations

Publication of this article was supported by the Canada Research Chair in Mathematical Genomics.

This article has been published as part of *BMC Bioinformatics *Volume 14 Supplement 15, 2013: Proceedings from the Eleventh Annual Research in Computational Molecular Biology (RECOMB) Satellite Workshop on Comparative Genomics. The full contents of the supplement are available online at http://www.biomedcentral.com/bmcbioinformatics/supplements/14/S15.
